# Care of the Teeth

**Published:** 1897-05

**Authors:** 


### Care of the Teeth.

These directions taken from the *Family Doctor* are evidently written by a practical man.

In addition to the use of a suitable tooth-brush and tooth-powder on the teeth, there is no practice which commends itself so highly as the use of a piece of silk thread. It will take the average person some time to become expert in handling it, but when this is attained, it will be acknowledged the best tooth-pick and beautifier of teeth in the world. Cut off from the reel a piece of silk about fifteen inches long, which thoroughly wax. With the thumbs and forefingers carry the waxed floss silk into each space between the teeth, the remaining three fingers of each hand being used to hold on to the ends of the silk firmly. The thumbs and forefingers of each hand as they hold the silk should be kept but a very little farther apart than the width of the teeth between which the silk is to be passed. Thorough tension of the silk must be kept up at all times. For the eight teeth on the left side of the upper jaw, pass the silk over the end of the left-hand thumb, and over the end of the right-hand forefinger. Thus the palm of the right hand and the back of the thumb of the left hand will be toward the face. Hold firmly, slide it between the teeth with a gliding motion; carry it well down between the necks of the teeth and the free edges of the gums, but not in such a manner as to wound the latter, the pressure being propeıly brought against the teeth, not against the gums. Before sliding the silk from between the teeth, the silk may be rapidly drawn backward and forward on the necks of the teeth, thus polishing and preserving these surfaces, and "raking out" any deposits of food or incipient tartar which may be there. The silk should be slid from between the teeth with the same tension as when it is introduced between them, otherwise it will tear when the teeth are very close together. If this rule be observed, and the silk still tears, it indicates one of several conditions—a cavity of decay; a scale of tartar; or a sharp point or jagged edge of the tooth, any of which conditions should be corrected by a reliable dentist.

